# Supplementary material for: Masticatory index for patients wearing dental prosthesis as alternative to conventional masticatory ability measures
Source: PLoS One. 2022 Jan 26;17(1):e0263048. doi: 10.1371/journal.pone.0263048 (PMC8791480; doi:10.1371/journal.pone.0263048)
Supplement: S1 Table — (DOCX) [file pone.0263048.s001.docx]

**S1 Table. Criteria for evaluating the retention and stability of removable partial dentures (RPD) as modified from the CU-modified Kapur index and NHANES III.**

| **Retention** | |
| --- | --- |
| 3 (maximum) | Maximum retention to vertical pull along path of insertion and lateral force (>10 N for dislodgement) |
| 2 (moderate) | Moderate retention to vertical pull along path of insertion and lateral force (5 – 10 N for dislodgement) |
| 1 (minimum) | Minimum retention to vertical pull along path of insertion and lateral force (<5 N for dislodgement) |
| 0 (no) | Displaced itself when seated |
| **Stability** | |
| 2 (Sufficient) | No movement of occlusal rests or indirect retainers when applied horizontal force to one- or both sides of the denture |
| 1 (Some) | Slight movement of occlusal rests or indirect retainers when applied horizontal force to one- or both sides of the denture |
| 0 (No) | Extreme visible movement of occlusal rests or indirect retainers when applied horizontal force to one- or both sides of the denture |
