# Supplementary material for: Masticatory index for patients wearing dental prosthesis as alternative to conventional masticatory ability measures
Source: PLoS One. 2022 Jan 26;17(1):e0263048. doi: 10.1371/journal.pone.0263048 (PMC8791480; doi:10.1371/journal.pone.0263048)
Supplement: S2 Table — (DOCX) [file pone.0263048.s002.docx]

**S2 Table. Criteria for evaluating the clinical quality of removable complete and partial dentures (modified from CU-modified Kapur)**

|  | Retention (score 0 to 3) | Stability (score 0 to 2) |
| --- | --- | --- |
| Acceptable maxillary denture | ≥2 | 2 |
| Acceptable mandibular denture | ≥1 | 2 |
| Acceptable removable partial denture | Acceptable both maxillary and mandibular removable partial denture | |
