# Supplementary material for: Masticatory index for patients wearing dental prosthesis as alternative to conventional masticatory ability measures
Source: PLoS One. 2022 Jan 26;17(1):e0263048. doi: 10.1371/journal.pone.0263048 (PMC8791480; doi:10.1371/journal.pone.0263048)
Supplement: Attachment — Submitted filename: Response to reviewers.docx [file pone.0263048.s004.docx]

**Response to reviewer**

The authors are pleased to submit our revised manuscript ID. PONE-D-21-02795, entitle ‘Masticatory Index for Patients Wearing Dental Prosthesis as Alternative to Conventional Masticatory Ability Measures’. The requested revisions have been made in the manuscript in track changes, and our point-by-point responses are below.:

1. Comment: Though the topic is of general interest I really do not see what new aspects relevant to the international scientific community are presented.

Response: The present study demonstrated the concept of developing a single masticatory index using a food questionnaire for evaluating masticatory ability of patients with various oral and denture status. Several new aspects have been proposed as follows:

1) Food questionnaires developed in previous studies included only the regularly-consumed foods. In contrast, the present study suggested developing the index that comprises both regularly- and rarely-consumed foods due to chewing difficulty. In addition, the food types should vary in hardness and toughness, and include sticky, grainy, fibrous foods. This is to improve the ability of the masticatory index to discriminate the patients with different oral and dental prosthesis status, in other words, to improve the ‘discriminant validity’ of the index. The description has been written in the ‘Discussion’ section (Page 15, 1^st^ paragraph)

Note: Discriminant validity measures constructs that theoretically should not be highly related or correlated to each other. The measurement with high discriminant validity would be able to discriminate the variables or constructs that are not correlated to each other.

2) In previous studies, masticatory index was validated with either a conventional subjective or objective masticatory ability measure by determining their associations with the conventional measures, in other words, determining the ‘convergent validity’. The convergent validity indicates that the index is as appropriate as the conventional masticatory ability measure.

In contrast, the present study determined not only the ‘convergent validity’, but also the ‘discriminant validity’ of the index compared with the conventional subjective and objective measures using the multiple regression analyses (Table 3). The discriminant ability of the masticatory index in identifying different tooth loss severities and denture qualities helps determine whether a developed food questionnaire better reflects masticatory ability compared with the conventional subjective and objective measures. The description has been written in the ‘Discussion’ section (Page 16, 1^st^ paragraph).

Therefore, despite the food items may vary among cultures, the concept of questionnaire development in this study can be applied worldwide for other studies with different cultures or environments to create their own masticatory indicator to be used in routine clinical practice and population-based study. The description has been written in the ‘Discussion’ section (Page 17, 2^nd^ paragraph)

2. Comment: The authors developed a food questionnaire based to the reports of only sixty individuals where 40 % were provided with complete dentures and RPDs and only 10% FPD or dentate. This may be already decisively biased as it is known that especially patients with CDs tend to select food that they can mince.

Response:

2.1) Our pilot study at Phase I included 40 patients (15 CD, 15 RPD, 10 FPD and dentate). They were interviewed for the regularly-consumed and rarely-consumed foods. Then, the additional 20 patients (total = 60) were also interviewed. We found that the most regularly-consumed and rarely-consumed foods obtained from 40 and 60 patients were similar.

Therefore, the reason for using only 60 individuals because the food items would be similar to those obtained from more than 60 patients.

2.2) The CUMI comprised both regularly-consumed and rarely-consumed foods. The inclusion of only 10% FPD and 10% dentate was due to the following reasons:

- The inclusion of more removable dentures was because they reported a greater variety of food types and textures compared with FPD and dentate individuals. The wide variety of food type was due to the variation in denture quality and oral status among the RPD and CD wearers. The increased food varieties help improve discriminant validity of the masticatory index.

- This questionnaire consists of both regularly-consumed and rarely-consumed foods due to chewing difficulty. In addition, not only CD but also RPD, FPD and dentate were interviewed. Therefore, we ensure that a selective bias reported by patients was minimized.

3. Comment: I am not a biostatistician but I doubt the power calculation. I have never seen a power calculation with a power of 0,99 and alpha = 0.05 on the basis of only 110 subjects. Even more as the Stds are quite high.

Response: The F test for analysis of variance (ANOVA) was used to determine the effect of different means between three groups. A type of power analysis was a post hoc comparison test to compute the achieved power, given an alpha, sample size and effect size.

To calculate the effect size, the mean CUMI values of the three groups (CD = 98.1, RPD = 82.2, and FPD = 63.7) and the highest standard deviation (s.d.) value among three groups (17.0) were used. Then, an effect size of 0.7027 was calculated. Given that the effect size = 0.7027, an alpha = 0.05, and a total sample of 110 within 3 groups, the power of 99% was achieved. The relatively high power is due to the significant differences between the FPD and the CD group. The calculation was shown in the below figure.

4. Comment: As the food items are special at least for Asian food the approach is very limited on worldwide perspective. On the other hand, peanuts that the authors use as a comparison are more or less available worldwide.

Response: The food items in the present questionnaire are special for Asian food, however, it is considered for worldwide use because the Asian-living culture and Asian populations are prevalent worldwide. In addition, the present study aimed not only to develop the questionnaire, but also to propose a concept of developing a questionnaire for masticatory ability evaluation in patients wearing different types of dental prosthesis. The authors believe that the readers can apply the concept and protocol of the present study to develop their own questionnaires, to be used in other cultures and countries.

Although peanut mastication is worldwide used, it is not practical in routine dental practice and population-based study because it requires special equipment and time-consuming to perform (The description has been written in the ‘Introduction’ section, Page 4, 1^st^ Paragraph). The special equipment includes standard test sieves and vibrating sieve shaker. Peanut test is time consumption since it takes 2 days for evaluation process, from obtaining peanut particles comminuted by patients, drying peanut particles for 24 hours, and then analyzing the peanut particle size on the next day to obtain the result. Additional personal is also required to perform the evaluation process. Therefore, peanut mastication is practical only in clinical study or research, while the developed CUMI is more practical to be used in routine clinical practice and population-based study.

5. Comment: The different chewing difficulty levels claimed for the food items were never assessed and /or verified.

Response: The different chewing difficulty levels have never been assessed/verified by any special instruments or equipment. This was because chewing difficulty level is the outcome which includes mixed properties of the food, for example, hardness, toughness, stickiness, slipperiness, fibrousness, and so on. Therefore, no specific instrument can be used to verify the different chewing difficulty levels of the food items.

However, the index was verified by three experts in prosthodontics, and determined their agreement in grading levels of chewing difficulty for each food item.

6. Comment: With regard to the results, I really do not see when compared to the peanuts test. The statement that the developed masticatory index reflects the patients’ ability better the conventional subjective or objective methods is not supported by the data.

Response: The comparison between the CUMI, peanut test, and eating impact has been performed by using the multiple regression analyses to determine the ‘discriminant validity’ of each measure (Table 3). The results showed that after adjusting for age and sex, there was a significant dose-response relationship between an increased CUMI score, greater tooth loss severity, and unacceptable denture quality. Meanwhile, a dose-response relationship was not shown in the peanut particle size and eating impact models. For type of dental prosthesis model, the peanut particle size was significantly different only between FPD and CD, whereas the oral impact was different only between the acceptable and unacceptable denture quality. For both oral and denture status models, the adjusted R^2^ values of the CUMI outcome was the highest, followed by those of peanut particle size and eating impacts. (The descriptions have been revised in the ‘Result’ section, Page 12).

The statistically significant differences in the CUMI score between different tooth loss severity, as well as type and quality of dental prosthesis indicated that the discriminant validity of the CUMI in identifying different oral and denture status was better than those of eating impacts and masticatory performance. The highest adjusted R^2^ values of the CUMI models, followed by those of the masticatory performance and eating impacts, indicated that oral and denture status better explained the variances in the CUMI score compared with masticatory performance and eating impacts. The descriptions have been written in the ‘Discussion’ section, Page 16, 1^st^ Paragraph.

Sincerely yours,

Wacharasak Tumrasvin

Corresponding author
