# Supplementary material for: Masticatory index for patients wearing dental prosthesis as alternative to conventional masticatory ability measures
Source: PLoS One. 2022 Jan 26;17(1):e0263048. doi: 10.1371/journal.pone.0263048 (PMC8791480; doi:10.1371/journal.pone.0263048)
Supplement: Attachment — Submitted filename: Response to reviewers_revise2.docx [file pone.0263048.s005.docx]

**Response to reviewers**

The authors are pleased to submit our revised manuscript ID. PONE-D-21-02795R1, entitle ‘Masticatory Index for Patients Wearing Dental Prosthesis as Alternative to Conventional Masticatory Ability Measures’. The requested revisions have been made in the manuscript in track changes, and our point-by-point responses are below.

**Reviewer #1:**

The authors would like to apologize for our misunderstandings regarding the first revision of the manuscript. Therefore, we have added the descriptions in the second revised version of the manuscript according to your recommendations.

1. Comment: The authors developed a food questionnaire based to the reports of only sixty individuals where 40 % were provided with complete dentures and RPDs and only 10% FPD or dentate. This may be already decisively biased as it is known that especially patients with CDs tend to select food that they can mince.

Response:

Initially, 40 patients (37.5% CD, 37.5% RPD, and 10% FPD and dentate) were asked about the typical food types they had eaten during the past week, and the food types they had difficulty in chewing or would like to eat but could not chew at all. Then, the additional 20 patients were interviewed. We found that the most regularly-consumed and rarely-consumed foods obtained from 40 and 60 patients were similar. Therefore, the Phase I comprised a total of 60 patients (40% CD, 40% RPD, 10% FPD and 10% dentate individuals). A higher proportion of removable dentures was included because they reported a greater variety of food types and textures compared with FPD and dentate individuals. The wide variety of food type was due to the variation in denture quality and oral status among the RPD and CD wearers. Then, 80 food types were obtained from the interview. The increased food varieties help improve discriminant validity of the masticatory index. These descriptions have been added in the ‘Materials and Methods’ section, ‘Phase I’ subsection (Page 6).

2. Comment: As the food items are special at least for Asian food the approach is very limited on worldwide perspective. On the other hand, peanuts that the authors use as a comparison are more or less available worldwide.

Response: The food items in the present questionnaire are special for Asian food, however, it is considered for worldwide use because the Asian-living culture and Asian populations are prevalent worldwide. In addition, the present study aimed not only to develop the questionnaire, but also to propose a concept of developing a questionnaire for masticatory ability evaluation in patients wearing different types of dental prosthesis. The authors believe that the readers can apply the concept and protocol of the present study to develop their own questionnaires, to be used in other cultures and countries. These descriptions have been added in the ‘Discussion’ section, 1^st^ Paragraph, Page 19.

Although multiple sieve method of peanut mastication is worldwide used, it consumes more time and resources to perform than the subjective measures. The whole evaluation process takes 2 days to obtain the result since collecting the comminuted peanut particles from patients, drying the comminuted peanut particles overnight, and then, analyzing the peanut particle size on the next day. Therefore, peanut mastication might be practical only in clinical study or research. These descriptions have been added in the ‘Discussion’ section, 1^st^ Paragraph, Page 17.

3. Comment: The different chewing difficulty levels claimed for the food items were never assessed and /or verified.

Response: The chewing difficulty levels were obtained from participants’ response, and the food items were ranked from the highest to lowest masticatory score. In addition to the participants’ responses, three experts in prosthodontics scored the masticatory difficulty grade of each food item, and the values were compared with those obtained from all participants. Weighted Kappa scores ranging from 0.75–0.87 were calculated, indicating 90–95% agreement between the experts and participants. These descriptions have been added in the ‘Materials and Methods’ section, ‘Phase II - CUMI assessment’ subsection (Page 8).

The different chewing difficulty levels were verified between dental experts and patients. However, we did not use any specific instrument to assess masticatory difficulty level of each food item because it includes mixed properties of the food such as hardness, toughness, stickiness, slipperiness, and fibrousness. Therefore, no specific instrument can comprehensively determine these properties and verify the outcome. These descriptions have been written in the ‘Discussion’ section, 2^nd^ Paragraph, Page 16.

4. Comment: The statement that the developed masticatory index reflects the patients’ ability better the conventional subjective or objective methods is not supported by the data.

Response: The developed masticatory index reflects the patients’ ability better than the conventional subjective and objective methods was supported by discriminant validity as shown by a dose-response relationship between the decreased CUMI score and increased tooth loss severity, and unacceptable denture quality. To support the statement, the revisions have been made in the ‘Abstract’ and ‘Discussion’ sections as follows:

- In ‘Abstract section’, the revisions regarding discriminant validity have been made in the Methods, Results, and Conclusion.
- In ‘Result’ section’ (Page 14), the revision has been made in the multiple regression analysis of the CUMI stated that “After adjusting for age and sex, there was a significant dose-response relationship between an increased CUMI score, greater tooth loss severity, and unacceptable denture quality. Meanwhile, a dose-response relationship was not shown in the peanut particle size and eating impact models. For type of dental prosthesis model, the peanut particle size was significantly different only between FPD and CD, whereas the oral impact was different only between the acceptable and unacceptable denture quality. For both oral and denture status models, the adjusted R^2^ values of the CUMI outcome was the highest, followed by those of peanut particle size and eating impacts. Therefore, the CUMI demonstrated better discriminant validity than the peanut particle size and eating impact models.”
- In ‘Discussion’ section, 1^st^ paragraph: “Since significant dose-response relationships were reported only between an increased CUMI score and greater tooth loss severity, and unacceptable denture quality, the CUMI demonstrated better discriminant validity than the conventional subjective and objective measures. The results indicated that the developed masticatory index better reflected patients’ masticatory ability compared with conventional subjective and objective measures.”

**Reviewer #2:**

1. Comments: The study design using specific Asian food makes the study hard to reproduce or repeat.

Response: The food items in the present questionnaire are special for Asian food, however, it is considered for worldwide use because the Asian-living culture and Asian populations are prevalent worldwide. In addition, the present study aimed not only to develop the questionnaire, but also to propose a concept of developing a questionnaire for masticatory ability evaluation in patients wearing different types of dental prosthesis. These descriptions have been added in the ‘Discussion’ section, 1^st^ Paragraph, Page 19.

2. Comments: The previous comments were not responded reasonably especially comment 2, 4 and 5.

Response: The authors have responded to the previous comments especially 2, 4 and 5 and have revised them in the manuscript according to the reviewer’s recommendations.
3. Comments: Is there any positive control in this study? please discuss.

Response: In this study, the FPD group (dentate individuals who had at least 26 remaining natural teeth) served as a positive control because they showed the least frequent eating impact, and the highest masticatory performance and CUMI score. Approximately 79% of them could easily chew all food items or get a full CUMI score. The descriptions have been added in the ‘Discussion’ section (2^nd^ Paragraph, Page 18) and mentioned in the “Materials and Methods” section.

Sincerely yours,

Wacharasak Tumrasvin

Corresponding author
