# Supplementary material for: Masticatory index for patients wearing dental prosthesis as alternative to conventional masticatory ability measures
Source: PLoS One. 2022 Jan 26;17(1):e0263048. doi: 10.1371/journal.pone.0263048 (PMC8791480; doi:10.1371/journal.pone.0263048)
Supplement: Attachment — Submitted filename: Response to reviewers_revise3.docx [file pone.0263048.s006.docx]

**Response to reviewers**

The authors would like to give thanks for your time spent in peer-reviewing our manuscript. We are pleased to submit our revised manuscript ID. PONE-D-21-02795R2, entitle ‘Masticatory Index for Patients Wearing Dental Prosthesis as Alternative to Conventional Masticatory Ability Measures’. The requested revisions have been made in the manuscript in track changes, and our point-by-point responses are below.

**Reviewer #3:**

1. Comment: The authors did a good job in addressing previous reviews and comments. The following are suggested modifications so that the manuscript can be acceptable for publication.

Response: Thank you for your feedback and comments.

2. Comment: In the introduction, authors keep using the term "dental prosthesis" which is a little bit confusing as they cite articles that report on complete dentures. Please unify terminology through the introduction and manuscript.

Response: A terminology of ‘dental prosthesis’ in this manuscript context has been defined in the 1^st^ paragraph of ‘Introduction’ section; “In this context, a dental prosthesis refers to fixed partial denture (FPD), removable partial denture (RPD), and complete denture (CD).” The references have been revised throughout the manuscript to ensure that the cited articles cover the term ‘dental prosthesis’.

3. Comment: Add a paragraph describing the limitations of this study and the drawn conclusions especially considering the subjects number.

Response: The limitations of this study has been written in a separated paragraph. The limitation, considering the number of participants, has been added in the last paragraph of the ‘Discussion’ section, and in the ‘Conclusion’ section.

Sincerely yours,

Wacharasak Tumrasvin

Corresponding author on behalf of all authors
